# Computational Discovery of Cancer Immunotherapy Targets by Intercellular CRISPR Screens

**DOI:** 10.3389/fimmu.2022.884561

**Published:** 2022-05-16

**Authors:** Soorin Yim, Woochang Hwang, Namshik Han, Doheon Lee

**Affiliations:** ^1^ Department of Bio and Brain Engineering, Korea Advanced Institute of Science and Technology (KAIST), Daejeon, South Korea; ^2^ Bio-Synergy Research Center, Daejeon, South Korea; ^3^ Milner Therapeutics Institute, University of Cambridge, Cambridge, United Kingdom; ^4^ Cambridge Centre for AI in Medicine, Department of Applied Mathematics and Theoretical Physics, University of Cambridge, Cambridge, United Kingdom

**Keywords:** intercellular interactions, ligand-receptor interactions, cell-cell communication, target discovery, immune checkpoint inhibitors, genome-wide CRISPR screen, triple-negative breast cancer, cytotoxic T cells

## Abstract

Cancer immunotherapy targets the interplay between immune and cancer cells. In particular, interactions between cytotoxic T lymphocytes (CTLs) and cancer cells, such as PD-1 (*PDCD1*) binding PD-L1 (*CD274*), are crucial for cancer cell clearance. However, immune checkpoint inhibitors targeting these interactions are effective only in a subset of patients, requiring the identification of novel immunotherapy targets. Genome-wide clustered regularly interspaced short palindromic repeats (CRISPR) screening in either cancer or immune cells has been employed to discover regulators of immune cell function. However, CRISPR screens in a single cell type complicate the identification of essential intercellular interactions. Further, pooled screening is associated with high noise levels. Herein, we propose intercellular CRISPR screens, a computational approach for the analysis of genome-wide CRISPR screens in every interacting cell type for the discovery of intercellular interactions as immunotherapeutic targets. We used two publicly available genome-wide CRISPR screening datasets obtained while triple-negative breast cancer (TNBC) cells and CTLs were interacting. We analyzed 4825 interactions between 1391 ligands and receptors on TNBC cells and CTLs to evaluate their effects on CTL function. Intercellular CRISPR screens discovered targets of approved drugs, a few of which were not identifiable in single datasets. To evaluate the method’s performance, we used data for cytokines and costimulatory molecules as they constitute the majority of immunotherapeutic targets. Combining both CRISPR datasets improved the recall of discovering these genes relative to using single CRISPR datasets over two-fold. Our results indicate that intercellular CRISPR screens can suggest novel immunotherapy targets that are not obtained through individual CRISPR screens. The pipeline can be extended to other cancer and immune cell types to discover important intercellular interactions as potential immunotherapeutic targets.

## 1 Introduction

Tumor immunotherapy invigorates the immune system to fight against cancer. It impedes interactions between immune cells, most notably cytotoxic T lymphocytes (CTLs), and cancer cells (1). For example, the well-known immune checkpoint inhibitor pembrolizumab is used to treat various types of cancer, including melanoma and triple-negative breast cancer (TNBC), as it targets PD-1 (*PDCD1*) and interferes with PD-L1 (*CD274*) interaction. Pembrolizumab prevents the PD-L1-induced suppression of CTL function, enabling cancer cell removal (2). However, immune checkpoint inhibitors are only effective in a subset of patients, with as little as 12.46% of cancer patients benefiting from such treatment in the United States (3). Therefore, novel immunotherapeutic targets and drugs are required.

Genome-wide clustered regularly interspaced short palindromic repeats (CRISPR) screens have been increasingly used for the systematic discovery of targets for cancer therapy. The most notable examples come for targeted therapy, where a large consortium called the Cancer Dependency Map performed 1076 genome-wide CRISPR screens in 908 cell lines (4, 5). Genome-wide CRISPR screening is also employed for immunotherapy (6–17). For example, pooled CRISPR screening in immune or cancer cells has been performed to evaluate immune regulatory molecules and identify potential therapeutic targets. Dong et al. subjected CTLs to CRISPR screens in order to identify gene knockouts that promote *in vivo* tumor infiltration and *in vitro* degranulation (15). Further, Lawson et al. utilized CRISPR in cancer cells to identify regulators of CTL-mediated killing (8). 

**Figure 1:**
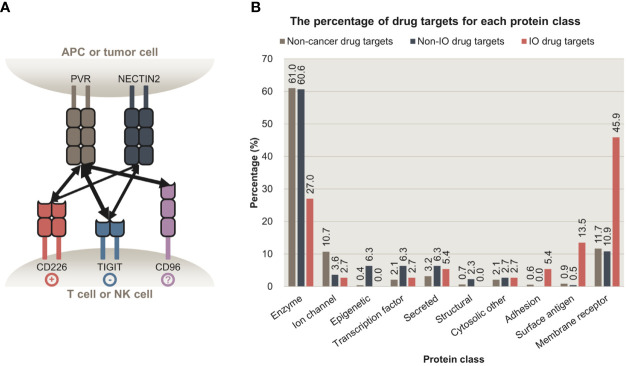
Intercellular interactions are potent targets for cancer immunotherapy. **(A)** Intercellular interactions between *PVR*, *NECTIN2*, *CD226*, *TIGIT*, and *CD96*. *PVR* and *NECTIN2* are expressed on antigen-presenting cells (APCs) and some tumor cells. Their receptors, *CD226*, *TIGIT*, and *CD96* are expressed on T cells or natural killer cells (NK cells). Upon binding to *PVR* or *NECTIN2*, *CD226* and *TIGIT* trigger stimulatory and inhibitory signals, respectively. Whether the binding of *PVR* to *CD96* delivers stimulatory or inhibitory signals is to be determined. The multiplicity of these interactions highlights the importance of focusing on intercellular interactions, rather than a single ligand or a receptor. **(B)** The percentage of proteins targeted by non-cancer, non-IO, and IO drugs that belong to each protein class. Membrane receptors, surface antigens, and adhesion proteins are preferentially targeted by IO drugs, relative to enzymes. APC, antigen-presenting cell; NK cell, natural killer cell; IO, immuno-oncology.

However, such CRISPR screening approaches have three main limitations. First, most studies did not focus on intercellular interactions, which underlie the mechanism of action for immunotherapy drugs, instead focused on single genes. Focusing on a single gene is suboptimal, as its protein product can interact with multiple partners (18). The drawbacks of this approach are evident when analyzing a ligand with opposing effects depending on the receptor bound (19). For example, *CD80* can activate or suppress T cells upon binding to *CD28* or *CTLA4,* respectively (20). Such competitive interaction was also observed among *PVR*, *CD226*, and *TIGIT,* (**Figure 1A**) (21). Moreover, our analysis revealed that a large proportion of immuno-oncology (IO) drugs (**Supplementary Figure 1**) and their targets (**Figure 1B**) are intercellular communication molecules such as adhesion molecules, surface antigens, and membrane receptors (**Supplementary Table 1**). These factors represent more robust drug targets than cytosolic proteins because they are exposed on the cellular membrane and are therefore more targetable (22). Second, studies have performed CRISPR screening using monocultured cells, even though monocultures do not recapitulate cell-cell communication (7). CRISPR screening under co-culture or other settings that potentially reflect intercellular interactions may also be limiting, as it is performed in a single cell type, i.e., in immune or cancer cells, which makes it difficult to pinpoint essential intercellular interactions, owing to the focus on a particular gene rather than on analyzing interactions (23). Lastly, genome-wide CRISPR screens inevitably entail high technical noise owing to their high throughput (24). To overcome these limitations and discover essential and potentially targetable intercellular interactions, CRISPR screens should be performed for all relevant interacting cell types.

In this study, we introduced intercellular CRISPR screens, a computational approach for the evaluation of intercellular interactions as IO drug targets. We employed intercellular CRISPR screening for the analysis of two genome-wide CRISPR datasets, an immune cell and a cancer cell dataset. Through integrating these datasets, we calculated the “intercellular normZ score” for each intercellular interaction and quantified its potential as a target for immunotherapy. As a proof of concept, we applied the intercellular CRISPR screen to two publicly available genome-wide CRISPR screening datasets, analyzing cancer cell and CTL interactions (8, 15). Our screens were more effective in identifying IO drug targets than CRISPR screens focusing on a single dataset.

## 2 Materials and Methods

### 2.1 Classification of Cancer Drugs and Their Targets

#### 2.1.1 Collection of Approved Cancer Drugs From DrugBank

The list of drugs, along with their development status (‘groups’), anatomical therapeutic chemical (ATC) codes, and target information was collected from DrugBank 5.1.8 (25). Among 14,585 drugs in DrugBank, 4207 belonged to the approved group, indicating that the drugs were approved in at least one jurisdiction at some time point. We set two criteria, either of which should be met for a drug to be termed a cancer drug; (1) The drugs should have ATC codes starting with ‘L01’ (antineoplastic agents), or (2) drugs belonging to the ‘Cancer immunotherapy’ category (accession number DBCAT005215) that were used in cancer treatment owing to their immunological effects. The DrugBank Clinical API was used to retrieve 72 approved drugs that belong to the ‘Cancer immunotherapy’ category. Altogether 221 cancer drugs were identified.

#### 2.1.2 Classification of Cancer Drugs Into IO *vs* Non-IO Using DrugBank and IO Landscape

Two resources were used to classify cancer drugs as IO and non-IO drugs. The first resource was the ‘Cancer immunotherapy’ category (accession number DBCAT005215) from DrugBank. The second was the IO Landscape, developed by the Cancer Research Institute to catalog the developmental status of IO drugs (26).

Among the ‘Cancer immunotherapy’ drugs from DrugBank, a few drugs such as trastuzumab, a *ERBB2* (HER2) inhibitor, are monoclonal antibodies that can be regarded as a targeted therapy rather than immunotherapy (27). However, a few monoclonal antibodies including pembrolizumab, a *PDCD1* inhibitor, are used as immunotherapeutic drugs. Therefore, we set two criteria, one of which should be met to classify a drug as an IO drug; (1) The drug belongs to the DrugBank ‘Cancer immunotherapy’ category and does not have ATC codes starting with ‘L01XC’ (monoclonal antibodies), or (2) A drug belongs to the ‘Cancer immunotherapy’ category, has ATC codes starting with ‘L01XC’, and is listed on IO Landscape. As a result, 47 cancer drugs were classified as IO drugs. The remaining 174 cancer drugs were classified as non-IO drugs.

#### 2.1.3 Protein Target Classification Using ChEMBL

Drug target information was collected from DrugBank. We used the classification term ‘targets’ and excluded ‘enzymes’, ‘transporters’, and ‘carriers’. As a result, 282 targets for 185 approved cancer drugs were identified.

The targets were classified using ChEMBL 29 (28). We used Level 1 classification terms to classify proteins into 14 categories. If a protein was classified as a “child” term, the term was mapped to the corresponding Level 1 terms based on the classification hierarchy. A target can have more than one category. For example, B-cell receptor CD22 was classified into the ‘Adhesion’, ‘Membrane receptor’, and ‘Surface antigen’ categories.

Drugs were categorized based on their target classification. If a drug had multiple targets, it was considered to belong to all classes into which its targets were classified.

### 2.2 Intercellular CRISPR Screens: Integration of CRISPR Screens in Immune and Cancer Cells to Prioritize Intercellular Interactions for IO Target Discovery

#### 2.2.1 Data Sources

We set five criteria for selecting CRISPR screening datasets in immune and cancer cells; (1) Genome-scale, and (2) publicly available datasets that were (3) obtained while the immune and cancer cells were interacting with each other. (4) The type of immune and cancer cells should be similar enough between the datasets and (5) transcriptome of the edited cell types should be available.

##### 2.2.1.1 Genome-Wide Pooled CRISPR Screens in CTLs

**Figure 2:**
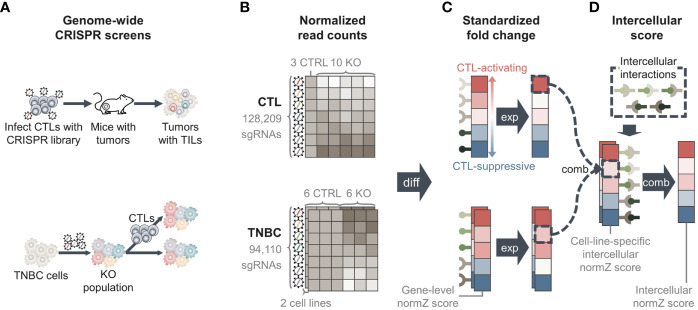
Overview of methods. **(A)** Data from two genome-wide pooled clustered regularly interspaced short palindromic repeats (CRISPR) screens were used. One CRISPR screen edited cytotoxic T lymphocytes (CTLs) to identify genes whose knockout increased the infiltration of CTLs into tumor tissue (upper) (15). The second screen edited two triple-negative breast cancer (TNBC) cell lines to identify genes whose knockout regulates the evasion of TNBC cells from CTL-mediated killing (lower) (8). **(B)** Genome-wide CRISPR screens yielded normalized read count matrices showing the amount of sgRNAs in each sample. TNBC CRISPR screen data had two matrices, one for each TNBC cell line. **(C)** We performed differential analysis to calculate fold changes of genes between knockout and control samples, yielding “Gene-level normZ scores”. Positive scores were assigned to genes that were more likely to activate CTL function, and negative scores were assigned to genes that were more likely to suppress CTL function. **(D)** We collected information for intercellular interactions from public databases and calculated the score of each intercellular interaction by ‘combining’ gene-level normZ scores of the interactants. Because two TNBC cell lines were used, two ‘cell-line specific intercellular normZ scores’ were obtained, one for each TNBC cell line. We combined cell-line-specific intercellular normZ scores to obtain the final intercellular normZ score. TIL, tumor-infiltrating lymphocyte; KO, knockout; CTRL, control; diff, differential analysis; exp, expression; comb, combination.

Genome-wide pooled CRISPR screens of CTLs were obtained from Dong et al. (15). The screen aimed to identify genes whose knockout increased the infiltration of CTLs into the tumor tissue *in vivo*. To achieve this goal, CTLs were transduced with a mouse genome-scale single guide RNA (sgRNA) library and were subsequently injected into *Rag1-/-* mice with TNBC, E0771 tumors (**Figure 2A**), and tumor tissues were harvested. The harvested tumor samples were compared with cellular libraries of infected CTLs that served as control samples to identify the enriched sgRNAs and genes in the tumor samples.

The normalized read count matrix was downloaded from **Table S1** (15). The dataset contained the normalized read counts of 128,209 sgRNAs targeting 21,786 genes from three cellular libraries and ten knockout samples (**Figure 2B**).

##### 2.2.1.2 Gene Expression in CTLs

In the study by Dong et al. (15), single-cell RNA sequencing was used to identify genes expressed in CD8+ tumor-infiltrating lymphocytes. From the paper, **Table S2** was used to obtain a list of genes expressed in CTLs as follows: First, low-quality cells with small library sizes (the number of unique molecule identifiers ≥4 standard deviations below the mean) or diversities (the number of detected genes ≥4 standard deviations below the mean), or large mitochondrial genes (the proportion of mitochondrial genes ≥4 standard deviations above the mean) were removed. Genes with low variance were regarded as being unexpressed. After converting Ensemble IDs into gene symbols, 7874 genes were identified expressed in CTLs (29).

##### 2.2.1.3 Genome-Wide Pooled CRISPR Screens in TNBC Cell Lines

A genome-wide pooled CRISPR screen in two mouse TNBC cell lines, 4T1 and EMT6, was conducted by Lawson et al. (8). The screening was performed to identify cancer genes whose knockout regulates the evasion of CTL-mediated killing of cancer cells. To this end, cancer cells were infected with the mouse Toronto knockout library and co-cultured with activated CTLs (**Figure 2A**). The number of sgRNAs in co-cultured cancer cells was compared with that in monocultured cancer cells as control samples. We downloaded two normalized read count matrices consisting of 94,110 sgRNAs targeting 19,459 genes in six co-culture and six monoculture samples for each cell line from **Supplementary Table 2** (8) (**Figure 2B**).

##### 2.2.1.4 Gene Expression in TNBC Cell Lines

To identify genes that were expressed in the mouse TNBC cell lines 4T1 and EMT6, bulk RNA sequencing data were obtained from **Supplementary Table 6** (8), from which the TNBC CRISPR screen data was collected. Two replicates were used for each TNBC cell line. A gene was considered as being expressed in a cell line if the mean expression level across replicates was at least ten fragments per kilobase of exon per million (FPKM) (30). If a gene had multiple probes, the probe with the highest average expression level was used. As a result, 6411 and 6518 genes were identified in 4T1 and EMT6, respectively.

##### 2.2.1.5 Intercellular Interactions From ConnectomeDB2020 and CellPhoneDB

Intercellular interaction data were collected from two sources: ConnectomeDB2020 (31) and CellPhoneDB (32). ConnectomeDB2020 contains 2293 manually curated ligand-receptor interactions. CellPhoneDB is a database of ligands, receptors, and describes 1396 interactions between these. Although both databases provide information on interaction data from humans, the CRISPR screens were performed in mouse cells. Therefore, we used orthologs to obtain potential ligand-receptor interactions in mice (29, 31). As a result, 4825 intercellular interactions between 1391 ligands and receptors were identified.

#### 2.2.2 Differential Analysis to Calculate Fold Change of Genes

We performed differential analysis using the drugZ algorithm (33) to identify genes that were significantly differentially enriched in knockout samples compared with control samples in each CRISPR dataset (**Figure 2C**). First, the drugZ algorithm compares sgRNA counts in knockout samples against control samples to calculate the fold changes in the amount of sgRNAs. Next, the algorithm standardizes the fold change by dividing it by the standard deviation and converts it into a *z*-score. To robustly estimate the standard deviation, drugZ uses empirical Bayes methods by borrowing information from sgRNAs with similar read counts in the control samples. Next, it combines the *z*-scores of individual sgRNAs targeting the same gene by summing the individual *z*-scores and dividing the sum by the square root of the number of summed sgRNAs. This yields the final normZ score, which follows a standard normal distribution.

For the TNBC CRISPR screening dataset, we used drugZ algorithm to calculate fold changes of genes co-cultured with CTLs compared to monocultured TNBC cells. As a result, genes whose knockout help TNBC cells escape from CTL-mediated killing obtained positive normZ scores. Since knockouts of these genes prevent CTLs from killing TNBC cells, they may be components of CTL-mediated cancer cell removal. For the CTL CRISPR screening dataset, we calculated the fold changes of genes in cell libraries against the tumor samples in order to give negative scores to CTL-suppressive genes. Genes whose knockout increased CTL infiltration into tumor tissue were enriched in the tumor sample, and therefore given negative scores. These genes may prevent CTL infiltration in their normal state. As a result, positive normZ scores were given to the genes that activate CTL function, while CTL-suppressive genes had negative normZ scores for both CRISPR screening datasets.

#### 2.2.3 Calculation of Intercellular normZ Score for Each Interaction

We combined two genome-wide CRISPR screening datasets to calculate the intercellular normZ scores. Before combining the CTL CRISPR and TNBC CRISPR screens, we set the normZ scores of unexpressed genes to zero for each cell type to reduce noise resulting from the high-throughput pooled screening procedure (5) (**Figure 2C**).

After zero out the normZ scores of unexpressed genes for each cell type, the normZ score of a ligand from one cell type and the corresponding receptor’s normZ score from the other cell type were summed to calculate the cell-line-specific intercellular normZ score (**Figure 2D** and **Supplementary Figure 2**). Because the gene-level normZ scores follow a standard normal distribution, we divided the sum by square root of two, i.e. the number of interacting genes, to obtain intercellular normZ scores that follow a standard normal distribution. As a result, we obtained two intercellular normZ scores, one each from 4T1-CTL and EMT6-CTL. For each intercellular interaction, we summed both normZ scores and divided the sum by the square root of two, i.e., the number of CTL-TNBC pairs, to obtain the final intercellular normZ score (**Figure 2D**). To obtain significant intercellular normZ scores, normZ scores from CTL, 4T1, and EMT6 CRISPR screens should have identical signs. If a ligand and a receptor obtained high normZ scores simply due to noise, there may be little chance that their normZ scores are large and have identical signs across all CTL, 4T1, and EMT6 CRISPR screens. Therefore, their normZ scores will offset each other during the summation, resulting in a small intercellular normZ score.

Because normZ scores follow the standard normal distribution (*z*-scores), the corresponding *p*-values and false discovery rates (FDRs) were calculated based on the normZ scores (33) (**Supplementary Table 2**).

#### 2.2.4 Alternative Methods to Calculate Intercellular normZ Scores

Other than the proposed method in 2.2.3, we devised two alternative methods to calculate the intercellular normZ scores. The first alternative method is ‘Strict’, which explicitly requires normZ scores to have the same sign when summed. If two normZ scores with different signs have to be summed, the ‘Strict’ method sets the result as zero instead of summing the scores. On the other hand, the proposed method in 2.2.3 does not put any constraints on the signs when two normZ scores are added, hence we named it as ‘Tolerant’. The second alternative method is ‘Composite’, a hybrid of ‘Strict’ and ‘Tolerant’. When the gene-level normZ scores of CTL and TNBC screens are summed, ‘Composite’ requires them to have the same sign. However, the intercellular normZ scores for 4T1-CTL and EMT6-CTL are not required to have the same sign to calculate the final score because cancer cell lines are highly heterogenous and they may not show the same immunological effect. In total, we used three different methods (‘Strict’, ‘Tolerant’, and ‘Composite’) to calculate intercellular normZ scores.

### 2.3 Collection of Well-Known Immunomodulators for Performance Evaluation

#### 2.3.1 Gold-Standard Dataset: Targets of Approved Drugs and Phase III Clinical Trial Drug Candidates for Immunotherapy

To benchmark the performance of intercellular CRISPR screens over using either of the screening data alone, a gold-standard dataset was obtained. Approved immunotherapy drugs that modulate CTLs were used as the gold-standard dataset to discover novel immunotherapeutic targets using CTL-related CRISPR screening data. We downloaded IO Landscape data and filtered drugs whose clinical stage was ‘Approved’ (26). Among 141 approved drugs, 97 drugs were classified as ‘T-cell targeted immunomodulators’ or ‘Other immunomodulators’. Among these, we retrieved 32 drugs whose target cell types were ‘APC/T cell’ or ‘T cell’. We manually inspected the 32 drugs and finalized the list of gold-standard drugs. However, only ten intercellular interactions were targeted by the approved drugs. Therefore, we obtained information for other drug candidates that modulate the function of CTLs and whose clinical stages were ‘Phase III’. As a result, 38 intercellular interactions between 47 genes and their effects on CTL function were identified (**Supplementary Table 3**).

#### 2.3.2 Silver Standard Dataset: Cytokines and Co-Stimulatory Molecules

The size of the gold-standard dataset was still limited to quantitatively evaluating and comparing the methods. Therefore, we collected a silver standard dataset composed of well-known immunomodulators as potential immunotherapeutic targets. These immunomodulators include cytokines, and co-stimulatory and co-inhibitory molecules since they compose the majority of targets of T cell modulators and other modulators (34). Because we used CRISPR datasets obtained while cancer cells and CTLs were interacting, we collected CTL-related immunomodulators only (20, 21, 35–52). As a result, we obtained 79 and 30 intercellular interactions known to activate and suppress CTL function, respectively.

### 2.4 Performance Comparison

#### 2.4.1 Aggregation of Intercellular normZ Scores Into Gene-Level Scores

Intercellular CRISPR screen evaluates each interaction, whereas CTL CRISPR and TNBC CRISPR screens evaluate each gene. To compare the intercellular CRISPR screen with the CTL and TNBC CRISPR screens, we aggregated interaction-level intercellular normZ scores into gene-level scores (**Supplementary Figure 3**). We enumerated all intercellular interactions involving each gene in CTL/TNBC cells. We hypothesized that strong interactions would result in intercellular normZ scores with high absolute values. Therefore, we used the intercellular normZ score with the highest absolute value as the score for the gene in the corresponding cell type.

#### 2.4.2 Performance Evaluation Metrics

We used the area under the receiver operating characteristic curve (AUROC), precision, recall, and F1 scores to evaluate the ability of CRISPR screens to discover the silver standard dataset. For the AUROC, we used the normZ scores. For precision, recall, and F1 score, we used FDR < 5% to classify normZ scores as CTL-activating, CTL-suppressive, and unknown. Genes/interactions with FDR ≥ 5% were classified as unknown. The remaining genes/interactions with positive and negative normZ scores were classified as CTL-activating and CTL-suppressive, respectively.

Because we have three classes, CTL-activating, CTL-suppressive, and unknown, we used an averaging scheme to evaluate performances. There are two averaging schemes in multiclass classifications: micro- and macro-averaging. Micro-averaging weighs each instance equally. On the other hand, macro-averaging regards each class as equally important, giving more weight to instances from minor classes. Since the silver standard dataset was highly imbalanced, we adopted the macro-averaging scheme. The performance for the unknown class was not evaluated since it may include potential immunotherapeutic targets that activate or suppress CTL functions.

## 3 Results

### 3.1 Intercellular CRISPR Screens Identify Approved Targets for Cancer Immunotherapy

To discover novel immunotherapeutic targets, we focused on intercellular interactions instead of single genes. We propose the use of intercellular CRISPR screens as a pipeline to discover potentially therapeutic interactions between immune and cancer cells. As a proof of concept, we used an intercellular CRISPR screen to identify the interactions that affect CTLs in TNBC (**Figure 2**). We used two genome-wide pooled CRISPR screen datasets that were collected while CTLs were interacting with TNBC cells (8, 15). Next, we collected intercellular interactions from CellPhoneDB and ConnectomeDB2020 (31, 32). By combining the two CRISPR screen datasets and the expression level of each gene in CTLs and TNBC cell lines, we calculated intercellular normZ scores, the extent to which each intercellular interaction affected CTL function. Intercellular normZ scores were calculated with three different methods. The first was ‘Strict’, which requires the immunological effects (CTL-activating or CTL-suppressive) of a ligand and a receptor should be concordant. Also, ‘Strict’ requires the immunological effects in different TNBC cell lines should be concordant. The second was ‘Tolerant’ since it permits a ligand and a receptor with different immunological effects. The last was ‘Composite’, a hybrid of ‘Strict’ and Tolerant. ‘Composite’ requires the effect of a ligand and a receptor to be concordant, whereas the effects in different TNBC cell lines can be different.

**Figure 3:**
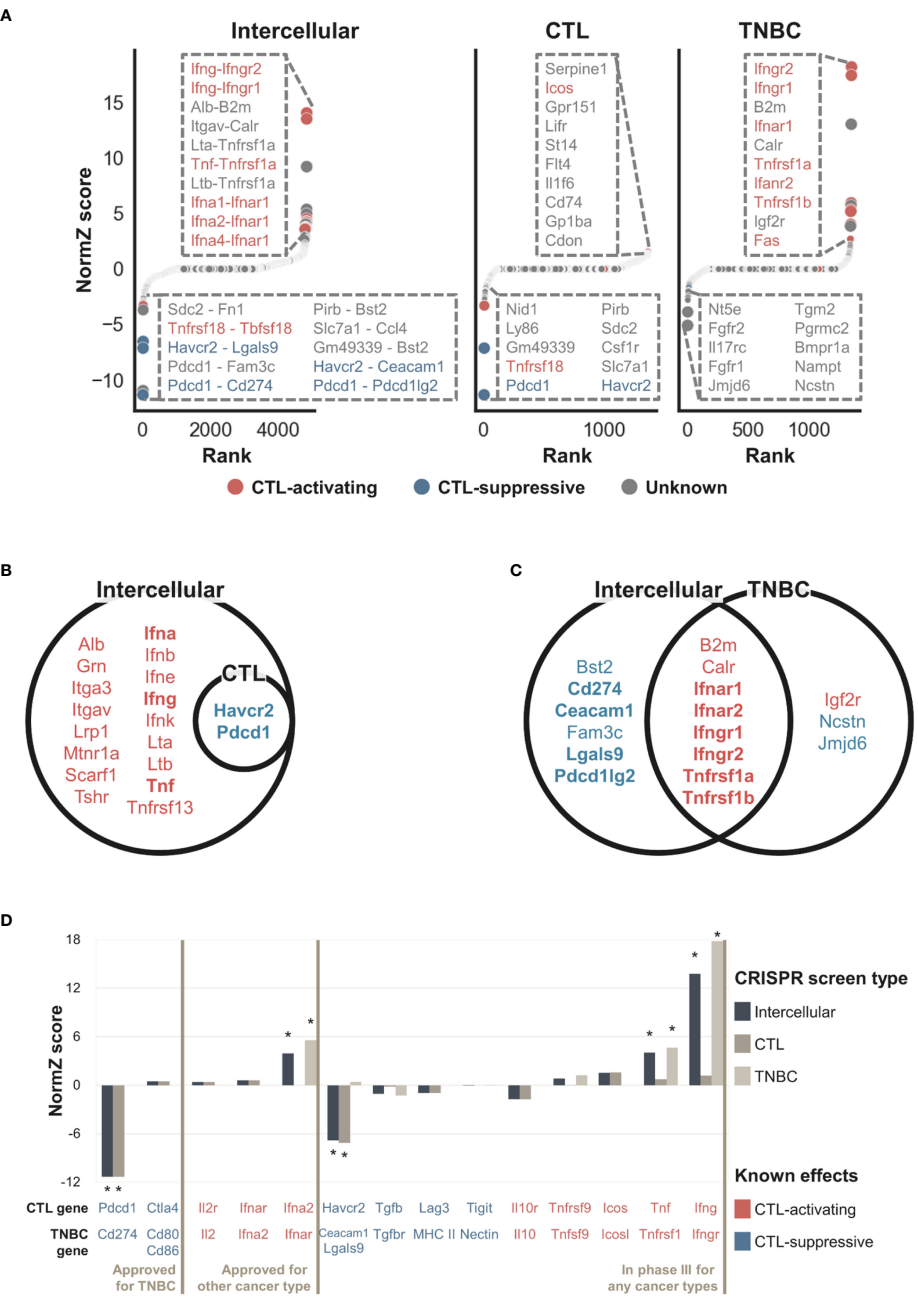
NormZ scores from the intercellular, CTL, and TNBC CRISPR screens. Intercellular normZ scores were calculated by the ‘Tolerant’ method. **(A)** Rank-ordered normZ scores from the intercellular (left), CTL (middle), and TNBC (right) CRISPR screens. Interactions/genes known to activate and suppress CTL function are marked in red and blue, respectively. The dot sizes are negatively proportional to the FDR. The top ten interactions/genes are represented in the inset. **(B)** Statistically significant (FDR < 5%) genes from the gene-level intercellular and CTL CRISPR screens. **(C)** Statistically significant (FDR < 5%) genes from the gene-level intercellular and TNBC CRISPR screens. Genes with positive and negative normZ scores are marked with red and blue, respectively. Well-known immunomodulators from the silver standard data are marked in bold. **(D)** The intercellular, CTL, and TNBC normZ scores of interactions/genes targeted by approved immunotherapeutic drugs, or phase III clinical trial drug candidates. * Statistically significant (FDR < 5%) interactions/genes.

**Figure 3A** shows that the intercellular CRISPR screen (‘Tolerant’ method) can identify both CTL-activating and suppressive interactions which can increase or inhibit CTL-mediated cancer cell removal, respectively. In contrast, the CTL and TNBC CRISPR screens are more suited for identifying CTL-suppressive and activating genes, respectively. The results can be explained as follows: knockout of CTL-activating genes decreases the number of CTLs, resulting in increased TNBC cell numbers. In contrast, the knockout of CTL-suppressive genes increases the number of CTLs, resulting in decreased TNBC cells. CTL CRISPR screens are more suitable for identifying genes that increase the number of CTLs, i.e., CTL-suppressive genes, whereas TNBC CRISPR screens are more suitable for identifying CTL-activating genes which can increase TNBC cell numbers. By combining both CRISPR screens, intercellular CRISPR screens can effectively identify CTL-activating and suppressive genes.

The intercellular CRISPR screen evaluates each interaction, whereas CTL and TNBC CRISPR screen evaluates a single gene. To directly compare intercellular CRISPR screen against CTL and TNBC ones, we aggregated intercellular normZ scores involving the identical gene to obtain gene-level intercellular normZ scores. We used FDR < 5% to predict genes as CTL-activating and CTL-suppressive based on the normZ scores. The results for gene-level comparisons showed a similar tendency (**Figure 3B, C**). The CTL CRISPR screen identified CTL-suppressive genes whereas genes discovered from TNBC CRISPR screens were biased towards CTL-activating ones. In contrast, intercellular CRISPR screens (‘Tolerant’ method) can help identify both CTL-activating and suppressive genes. For example, CTL-activating interactions between *Ifna*, *Ifng*, *Tnf*, and their corresponding receptors were not identifiable from CTL CRISPR screen, while intercellular CRISPR screen successfully discovered them (**Figure 3B** and **Supplementary Figure 4A**). Similarly, CTL-suppressive interactions *Pdcd1*-*Cd274, Havcr2*-*Ceacam1*, and *Havcr2*-*Lgals9* were identified by intercellular CRISPR screen, but not by TNBC CRISPR screen (**Figure 3C** and **Supplementary Figure 4B**). On the other hand, *Jmjd6* was identified by TNBC CRISPR screen, but not by intercellular CRISPR screen (**Figure 3C** and **Supplementary Figure 4B**). Even though *Jmjd6* was suggested to suppress the function of CTLs from *in vitro* TNBC CRISPR screen, it showed the opposite effect from *in vivo* CRISPR screens performed by Lawson et al. (8). This supports that intercellular CRISPR screen correctly rejected *Jmjd6*.

To benchmark the use of intercellular CRISPR screens (‘Tolerant’ method) relative to the use of either CRISPR screen alone, we obtained a gold-standard dataset containing data for 38 intercellular interactions targeted by approved IO drugs or phase III clinical trial drug candidates for immunotherapy. **Figure 3D** shows that CTL-suppressing and activating interactions tended to have negative and positive scores, respectively. Significant interactions were mostly related to cancer antigen presentation and killing of cancer cells, reflecting the experimental endpoint of the used CRISPR screens (53). This may be the reason why *Ctla4* obtained insignificant scores since it is related to the proliferation of CTLs in lymph nodes rather than killing cancer cells in peripheral tissue (53, 54). A few interactions were missed when CRISPR screen data were used alone. For example, the TNBC CRISPR screen failed to identify *Cd274* (Pd-l1) because it was not expressed in either 4T1 or EMT6 cells. However, this result is biologically plausible because not all patients express *PD-L1* or respond to anti-*PD-L1* therapy (55). In contrast, the CTL CRISPR dataset failed to identify interferons alpha and gamma. These results indicated that intercellular CRISPR screens combined the complementary CRISPR datasets to identify IO targets more comprehensively. However, a few gold-standard interactions had negligible scores. For example, aldesleukin is a recombinant interleukin-2 that has been approved for the treatment of renal cell carcinoma. However, the normZ scores of *Il2* and *Il2r* were close to zero in the CTL and TNBC CRISPR screens, respectively. Interestingly, we found that interleukin-2 failed to demonstrate its efficacy in breast cancer patients during a phase III clinical trial (56). Similarly, pegylated recombinant interleukin-10 or pegilodecakin had low efficacy against pancreatic ductal adenocarcinoma during a phase III clinical trial (57).

In summary, the intercellular CRISPR screen identified two intercellular interactions, *Pdcd1*-*Cd274* and *Ifna2*-*Ifnar* targeted by approved IO drugs pembrolizumab and peginterferon alfa-2b, respectively. In addition, our results suggested *Havcr2*-*Lgals9* and *Ifng*-*Ifngr*, targeted by sabatolimab and interferon gamma-1b respectively, as potentially therapeutic interactions. These results indicate that intercellular CRISPR screens can be used to discover effective targets during the early drug development stages.

### 3.2 Intercellular CRISPR Screens Outperform Single CRISPR Screens

We performed three quantitative evaluations to compare the performances of different CRISPR screens. First, we evaluated the performance of intercellular CRISPR screens calculated by the ‘Tolerant’ method. Next, we compared the ‘Tolerant’ method with two alternative methods, ‘Strict’ and ‘Composite’, to identify the best way to calculate intercellular normZ scores. Lastly, we compared the performance of intercellular CRISPR screens with CTL and TNBC CRISPR screens to estimate the degree of performance enhancement. Because there are few IO drugs, the gold-standard dataset may not be appropriate for the quantitative evaluation. Therefore, we used a silver standard dataset containing cytokines and co-stimulatory molecules as potential immunotherapeutic targets. Cytokines and co-stimulatory molecules were selected since they compose the majority of targets of immunomodulatory drugs (34). Because intercellular normZ scores are continuous, they were binarized based on an FDR < 5% to make predictions.

**Figure 4:**
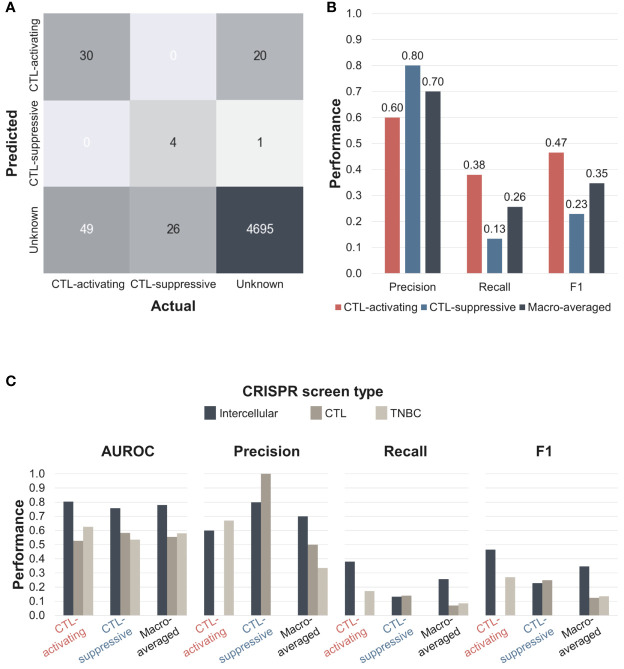
The performance of the intercellular CRISPR screen (‘Tolerant’ method). **(A)** Confusion matrix of intercellular CRISPR screens. Predictions were made based on an FDR < 5%. **(B)** Precision, recall, and F1 scores of intercellular CRISPR screens. **(C)** AUROC, precision, recall, and F1 scores of CTL, TNBC, and intercellular CRISPR screens. AUROC, area under the receiver operating characteristic curve.

The confusion matrix of the ‘Tolerant’ method is shown in **Figure 4A**. Among 79 CTL-activating interactions in the silver standard dataset, 30 were correctly classified as CTL-activating, whereas the remaining 49 were classified as unknown. Among 30 CTL-suppressive interactions, four were classified as CTL-suppressive, whereas the remaining 26 were classified as unknown. Even though 21 CTL-suppressive interactions obtained negative normZ scores, only four were statistically significant, i.e. FDR < 5%. Among 4716 interactions whose effects are unknown, 20 and one interaction were classified as CTL-activating and CTL-suppressive, respectively, and suggested as potential immunotherapeutic targets. Based on the confusion matrix, we evaluated the precision, recall, and F1 scores of intercellular CRISPR screen against the silver standard dataset. We used macro-averaging to deal with multiclass classification. Macro-averaged precision, recall, and F1 score were 0.70, 0.26, 0.35, respectively (**Figure 4B**). The recall was lower than precision since we used a strict threshold, FDR < 5%, for the classification to identify highly confident potential targets.

We evaluated two alternative methods to calculate the intercellular normZ scores (**Supplementary Figure 5**). The first method is ‘Strict’, which explicitly requires normZ scores to have the same sign when summed. We named the method in **Figure 2** as ‘Tolerant’ since it does not put any constraints on the signs of normZ scores. The second alternative method is ‘Composite’, which requires the gene-level normZ scores of CTL and TNBC screens to have the same sign. However, the intercellular normZ scores for 4T1-CTL and EMT6-CTL are not required to have the same sign under the ‘Composite’ method. We evaluated ‘Tolerant’, ‘Composite’, and ‘Strict’ when used with and without gene expression data. The best performance was obtained using the ‘Tolerant’ method with expression data, whereas the ‘Strict’ method with expression data performed the worst. For the ‘Composite’ and ‘Strict’ methods, the use of gene expression data reduced the performances due to the strict constraint on the normZ scores which resulted in many zeros. In contrast, including expression data improved the performance of the ‘Tolerant’ method. Therefore, we used the ‘Tolerant’ method with expression data to calculate the intercellular normZ scores.

Next, we compared the performance of the intercellular CRISPR screen relative to the CTL and TNBC CRISPR screens. We calculated the AUROC, precision, recall, and F1 scores for each CRISPR screen (**Figure 4C** and **Supplementary Figure 6**). The results showed that the intercellular CRISPR screen outperformed both individual CRISPR screens for all macro-averaged evaluation metrics. For class-specific performances, intercellular CRISPR screen achieved similar or superior performances except for the precision of the CTL and TNBC screen for CTL-suppressive and CTL-activating classes, respectively. However, the CTL and TNBC CRISPR screen failed to identify the opposite classes, resulting in impaired macro-averaged precision and recall. In fact, the intercellular CRISPR screen improved the macro-averaged recall and F1 score over single CRISPR screens more than twice. These results demonstrate that intercellular CRISPR screens can use complementary CRISPR screens to robustly identify immunotherapeutic targets.

### 3.3 Intercellular CRISPR Screens Identify Potential IO Targets

Based on the intercellular normZ scores, we identified potential IO targets with previously unknown effects. We investigated 21 interactions with FDR < 5% (**Figure 4A**), and additional three interactions whose absolute values of intercellular normZ scores were >3. We finalized seven interactions based on the following criteria; (1) Interactions whose effects from CTL and TNBC CRISPR screens were concordant, and (2) interactions involving genes expressed in CTLs. We included genes that were not expressed in TNBC cell lines because a few genes, including *CD274*, may be expressed in only a subset of TNBC cell lines.

Among the seven interactions identified, none was identifiable when CTL CRISPR screen was used only. On the other hand, *Calr*, *Tnfrsf1a*, and *Tnfrsf1b* were discovered from TNBC CRISPR screens while *Bst2*, *Ccl4*, and *Fn1* were not. Among them, six interactions were partially supported by literature (**Table 1**). Three interactions involved *Tnfrsf1a/b*, which activate CTL function (49). In particular, *Tnfrsf1b*-mediated activation of CTLs showed tumor regression in syngeneic EMT6 models (58). On the other hand, knockdown of *Bst2* increased tumor latency and reduced tumor volume in E0771 and 4T1 models (59). It is particularly interesting because *Bst2* was not identifiable from 4T1 CRISPR screen (FDR=0.543). In addition, a recent study reported that *Slc7a1* reduces memory T cells by activating mTORC1 (60), and *Sdc2* facilitates the removal of the T-cell receptor/CD3 complex from the cell membrane (61).

**Table 1:** Highly ranked intercellular interactions for immunotherapeutic targets.

Rank	CTL gene	TNBC gene	CTL		4T1		EMT6		Intercellular		Supporting literature
			normZ score	FDR	normZ score	FDR	normZ score	FDR	normZ score	FDR	
9	*Itgav*	*Calr*	1.33	0.546	4.28	0.003	3.81	0.011	5.375	4.62e-5	
10	*Lta*	*Tnfrsf1a*	1.19	0.546	2.49	0.552	5.05	7.5e-5	4.96	3.40e-4	(49)
11	*Ltb*	*Tnfrsf1a*	0.51	0.546	2.49	0.552	5.05	7.5e-5	4.28	0.003	(49)
30	*Lta*	*Tnfrsf1b*	1.19	0.546	0.96	0.552	4.74	2.53e-4	4.04	0.004	(49, 58)
56	*Gm49339*	*Bst2*	-2.72	0.613	-0.88	0.543	-1.04	0.548	-3.68	0.094	(59)
64	*Slc7a1*	*Ccl4*	-3.28	0.178	0.36*	0.543	-0.02*	0.544	-3.28	0.313	(60)
67	*Sdc2*	*Fn1*	-2.52	0.613	-0.91	0.543	-0.42	0.548	-3.185	0.388	(61)

None of the genes were identifiable from CTL CRISPR screens since their FDRs are higher than 0.05. Even though Calr, Tnfrsf1a, and Tnfrsf1b were statistically significant from TNBC CRISPR screens (FDR < 0.05), Bst2, Ccl4, and Fn1 were not. * Ccl4 was not expressed in 4T1 and EMT6 TNBC cell lines. All the other genes were expressed in the corresponding cell types.

In summary, our results suggested seven intercellular interactions as immunotherapeutic targets for TNBC. Among these, four may activate CTL function, for which agonists can be investigated as IO drugs. The remaining three were suggested to suppress CTL function, for which antagonists may be investigated to treat TNBC.

## 4 Discussion

In this study, we showed the utility of intercellular CRISPR screens for the discovery of immune–cancer cell interactions as IO targets, relative to focusing on single genes. Intercellular CRISPR screens integrate two CRISPR screens, one for immune and one for cancer cells, which are obtained when both cells interact with each other. Our results showed that CTL and TNBC CRISPR screens were complementary, and the intercellular CRISPR screen outperformed individual screens.

Although our results successfully identified approved IO targets and known immunomodulators, they have three limitations. First, the experimental settings for the CTL and TNBC CRISPR screens were different. The CTL and TNBC CRISPR screens were used *in vivo* and *in vitro*, respectively. In addition, the screens used different TNBC cell lines. Although we tried to find datasets with similar experimental conditions, there were few studies that satisfied our five selection criteria. We expect that using CRISPR screening datasets obtained under identical experimental conditions and incorporating more datasets would lead to more robust results. Second, public databases were used to identify putative interactions between CTLs and TNBC cells. Growing efforts have been made to infer cell-type-specific intercellular interactions by combining information from public databases with large-scale gene expression data (62). Although we set the scores of unexpressed genes to zero, adopting sophisticated methods to infer cell-type-specific intercellular interactions may refine intercellular CRISPR screens to help identify more specific targets. Lastly, potential IO targets in **Table 1** were not experimentally validated. Even though we listed some supporting literature, they should be validated by experiments.

Despite these limitations, the intercellular CRISPR screening method identified nine (**Figure 3** and **Supplementary Table 3**) and 34 interactions (**Figure 4A**) that are targeted by approved drugs and phase III clinical trial drug candidates, respectively. Moreover, our results suggested seven interactions as potential IO targets (**Table 1**). Focusing on interactions instead of single genes overcomes the limitation of not accounting for a single gene having multiple interaction partners. In addition, the novel method proposed in the study is useful because ligands and receptors are generally druggable. A recent study suggested that a PD-1 and PD-L1 bi-specific antibody is more effective than using anti-PD-1 and anti-PD-L1 antibodies together (63), further supporting the use of intercellular CRISPR screens over single screens.

Considering the aforementioned advantages, intercellular CRISPR screens can be used to evaluate interactions between several cancer and immune cell types such as natural killer cells or macrophages (23, 64). Moreover, the proposed method can be extended to consider multiple immune cell types simultaneously in order to more precisely model the tumor microenvironment.

## Data Availability Statement

The datasets presented in this study can be found in online repositories. The names of the repository/repositories and accession number(s) can be found in the article/**Supplementary Material**.

## Author Contributions

SY and WH conceptualized the study. SY developed and evaluated the proposed method along with WH under the supervision of NH and DL. All authors contributed to writing, reading, and have approved the manuscript.

## Funding

The work described and publication of this article were supported by the Bio-Synergy Research Project (NRF-2012M3A9C4048758) of the Ministry of Science and ICT through the National Research Foundation. NH and WH were funded by LifeArc.

## Conflict of Interest

NH is a cofounder of KURE.ai and CardiaTec Biosciences and an advisor at Biorelate, Promatix, Standigm, VeraVerse, and Cellaster.

The remaining authors declare that the research was conducted in the absence of any commercial or financial relationships that could be construed as a potential conflict of interest.

## Publisher’s Note

All claims expressed in this article are solely those of the authors and do not necessarily represent those of their affiliated organizations, or those of the publisher, the editors and the reviewers. Any product that may be evaluated in this article, or claim that may be made by its manufacturer, is not guaranteed or endorsed by the publisher.

## Abbreviations

CRISPR, clustered regularly interspaced short palindromic repeats; CTL, cytotoxic T lymphocyte; TNBC, triple-negative breast cancer; IO, immuno-oncology; ATC, anatomical therapeutic chemical; sgRNA, single guide RNA; TIL, tumor-infiltrating lymphocyte; KO, knockout; CTRL, control; diff, differential analysis; exp, expression; comb, combination; FDR, false discovery rate; AUROC, area under the receiver operating characteristic curve.
