# Supplementary material for: Computational Discovery of Cancer Immunotherapy Targets by Intercellular CRISPR Screens
Source: Front Immunol. 2022 May 16;13:884561. doi: 10.3389/fimmu.2022.884561 (PMC9149307; doi:10.3389/fimmu.2022.884561)
Supplement: Supplementary file 1 [file DataSheet_1.docx]

Supplementary Material

# Supplementary Figures and Tables

## Supplementary Figures


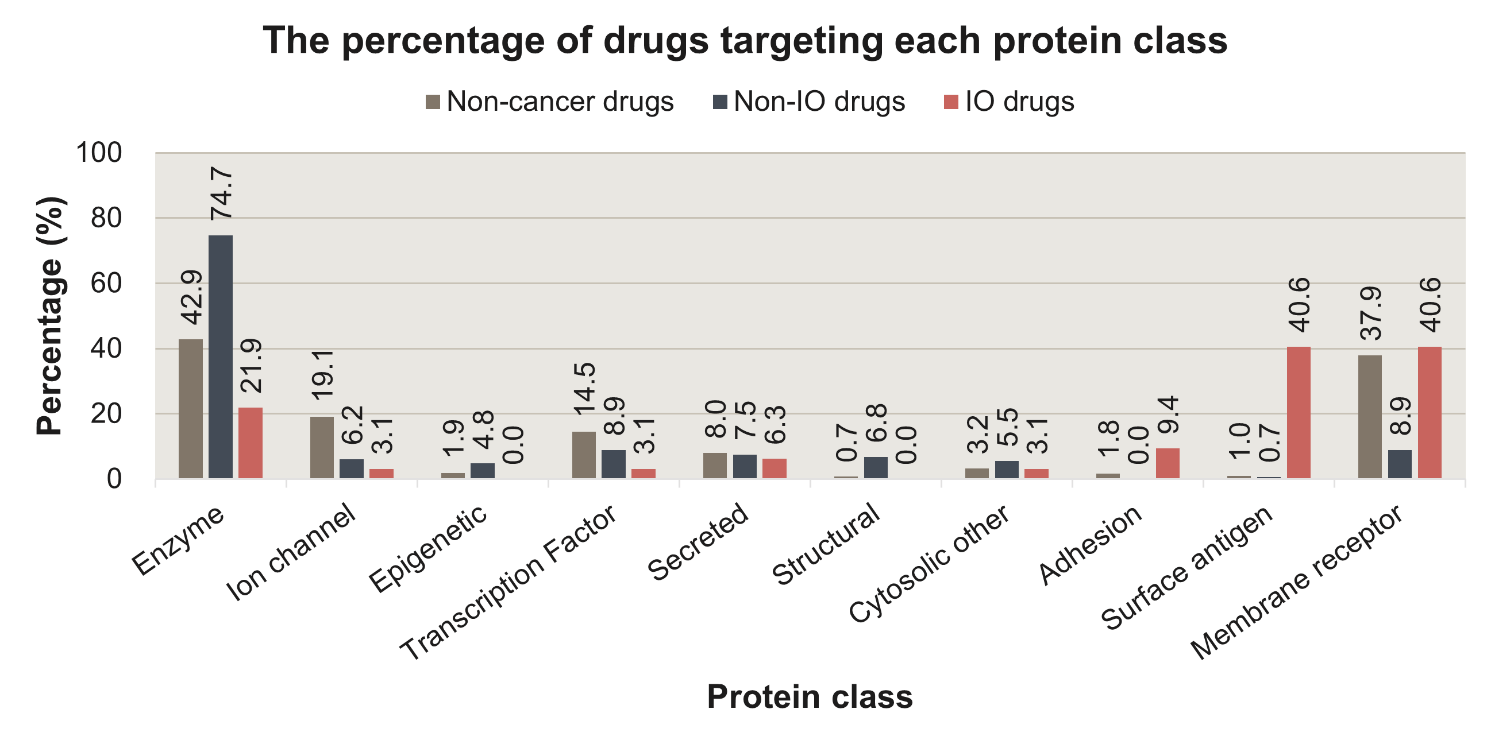


Supplementary Figure 1. The percentage of non-cancer and cancer drugs whose targets belong to each protein class. Cancer drugs were further categorized into non-immuno-oncology (non-IO) and IO drugs. Membrane receptors, surface antigens, and adhesion proteins are preferentially targeted by IO drugs, whereas enzymes are less favorably targeted by IO drugs. IO: immuno-oncology.


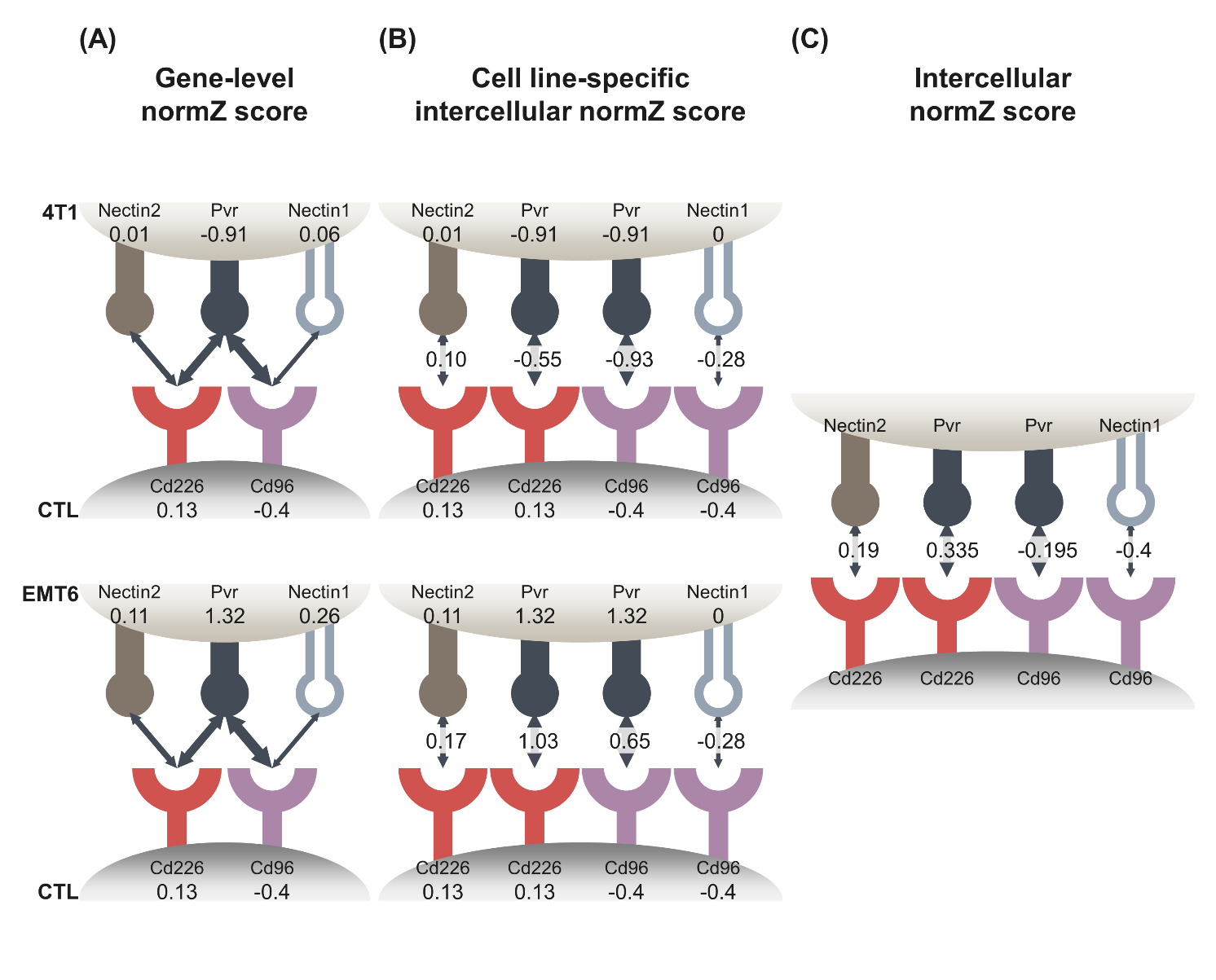
**Supplementary Figure 2.** An example for the calculation of intercellular normZ scores. **(A)** Gene-level normZ scores for CTLs and two TNBC cell lines, 4T1 and EMT6, were calculated by applying the drugZ algorithm to the normalized read count matrices. **(B)** Gene-level normZ scores of *Nectin1* in 4T1 and EMT6 were set to zero because *Nectin1* was not expressed in both TNBC cell lines. The cell line-specific intercellular normZ score was calculated by summing the gene-level normZ scores of the ligand and the receptor and dividing the sum by the square root of two. **(C)** The final intercellular normZ score was calculated by summing two cell line-specific intercellular normZ scores and dividing the sum by the square root of two. CTL: cytotoxic T lymphocyte.

**
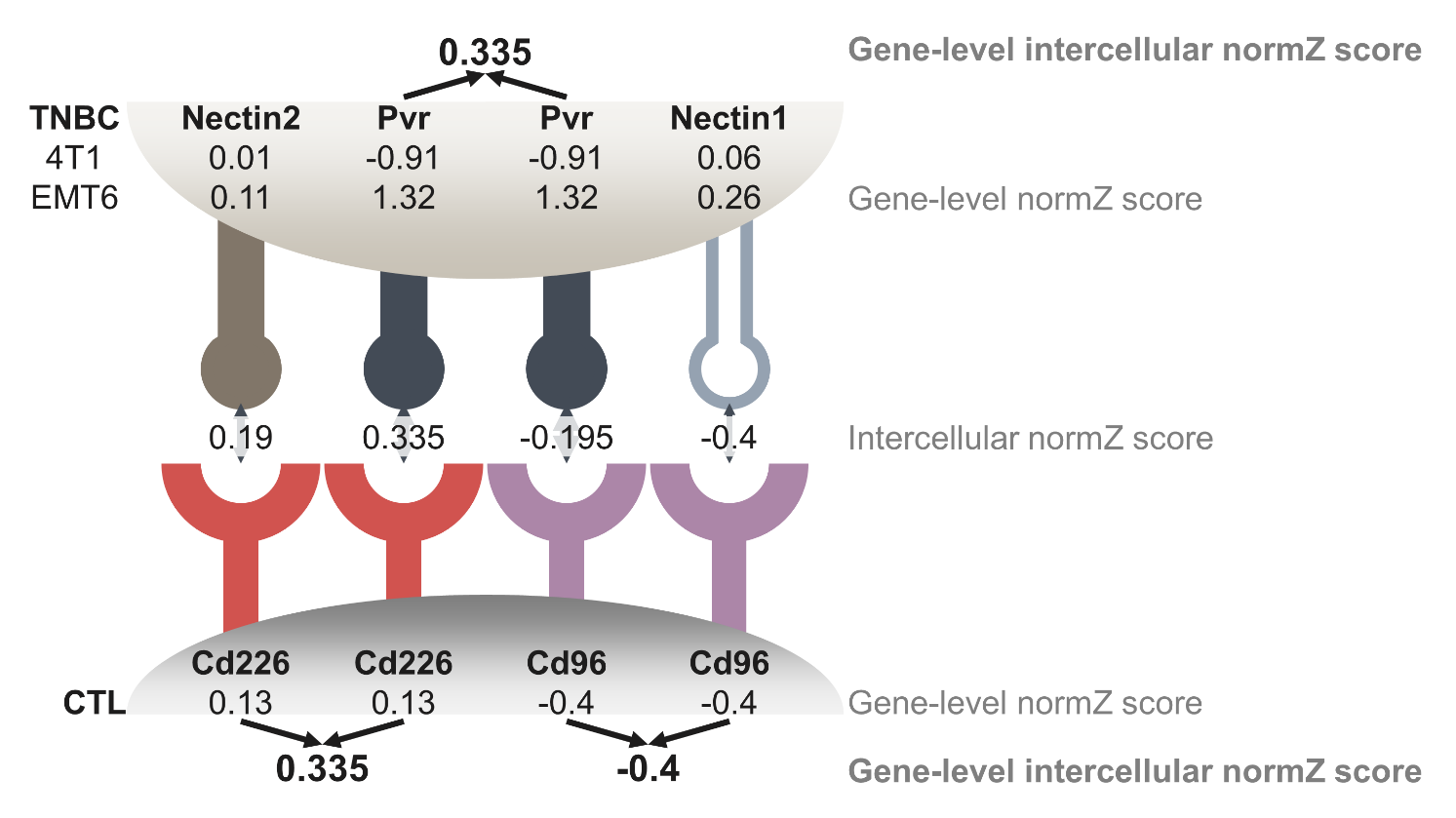
**

Supplementary Figure 3. An example for aggregating intercellular normZ scores into a gene-level score. Gene-level normZ scores of genes are indicated below the gene symbols. Intercellular normZ scores of interactions are indicated between ligands and receptors. Gene-level intercellular normZ scores for a gene in CTL/TNBC cells were obtained by taking the intercellular normZ score with the highest absolute value. TNBC: triple-negative breast cancer, CTL: cytotoxic T lymphocyte.


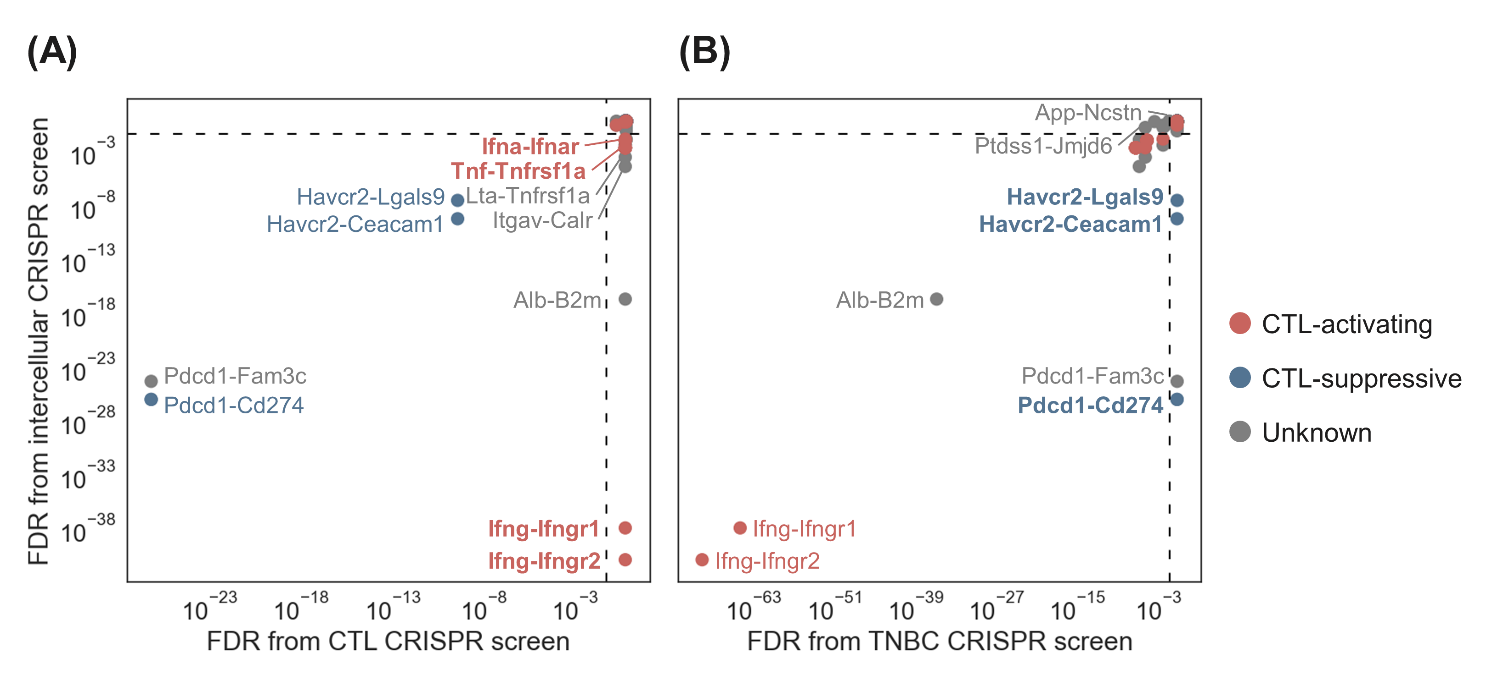


Supplementary Figure 4. The scatter plots of FDRs from intercellular CRISPR screens compared to CTL (A) and TNBC (B) CRISPR screens. The dashed lines represent FDR=0.05. Interactions in the upper left area were identifiable when CTL/TNBC CRISPR screens were used alone (FDR from intercellular CRISPR screen ≥ 0.05, FDR from CTL/TNBC CRISPR screen < 0.05), whereas interactions in the lower right area (FDR from intercellular CRISPR screen < 0.05, FDR from CTL/TNBC CRISPR screen ≥ 0.05) were identifiable from intercellular CRISPR screen only. Well-known immunomodulatory interactions in the silver standard dataset in the lower right area were represented as bold. FDR: false discovery rate, CTL: cytotoxic T lymphocyte. TNBC: triple-negative breast cancer


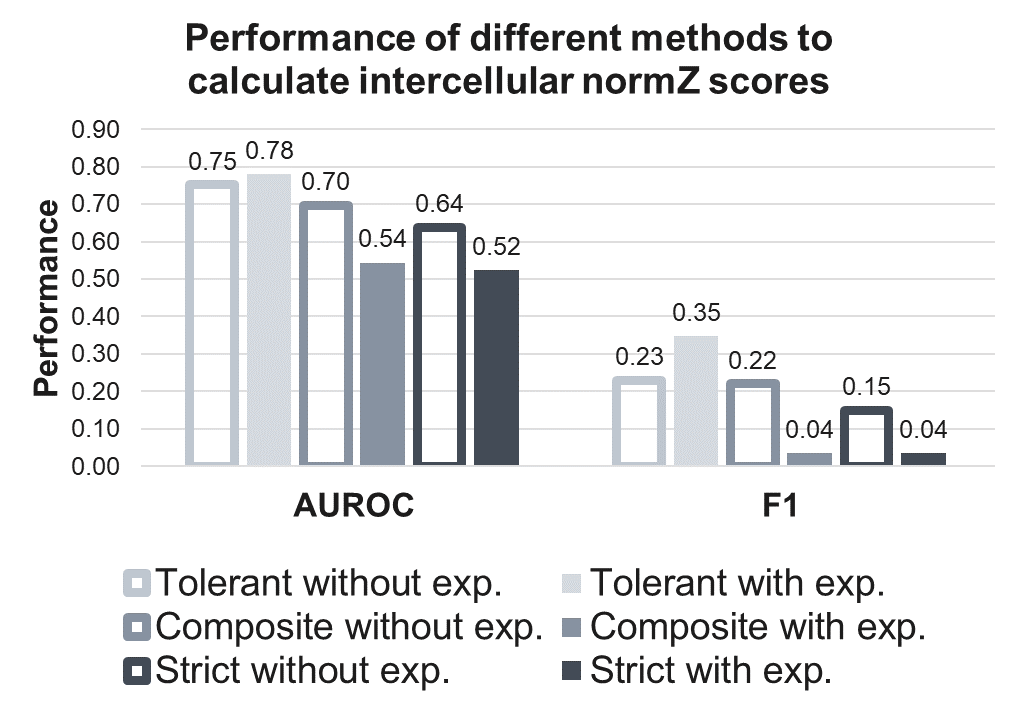


Supplementary Figure 5. Performance comparison of different methods to calculate intercellular normZ scores. “Tolerant” with expression data achieved the highest macro-averaged AUROC and F1 scores. AUROC: area under the receiver operating characteristic curve, exp.: expression.


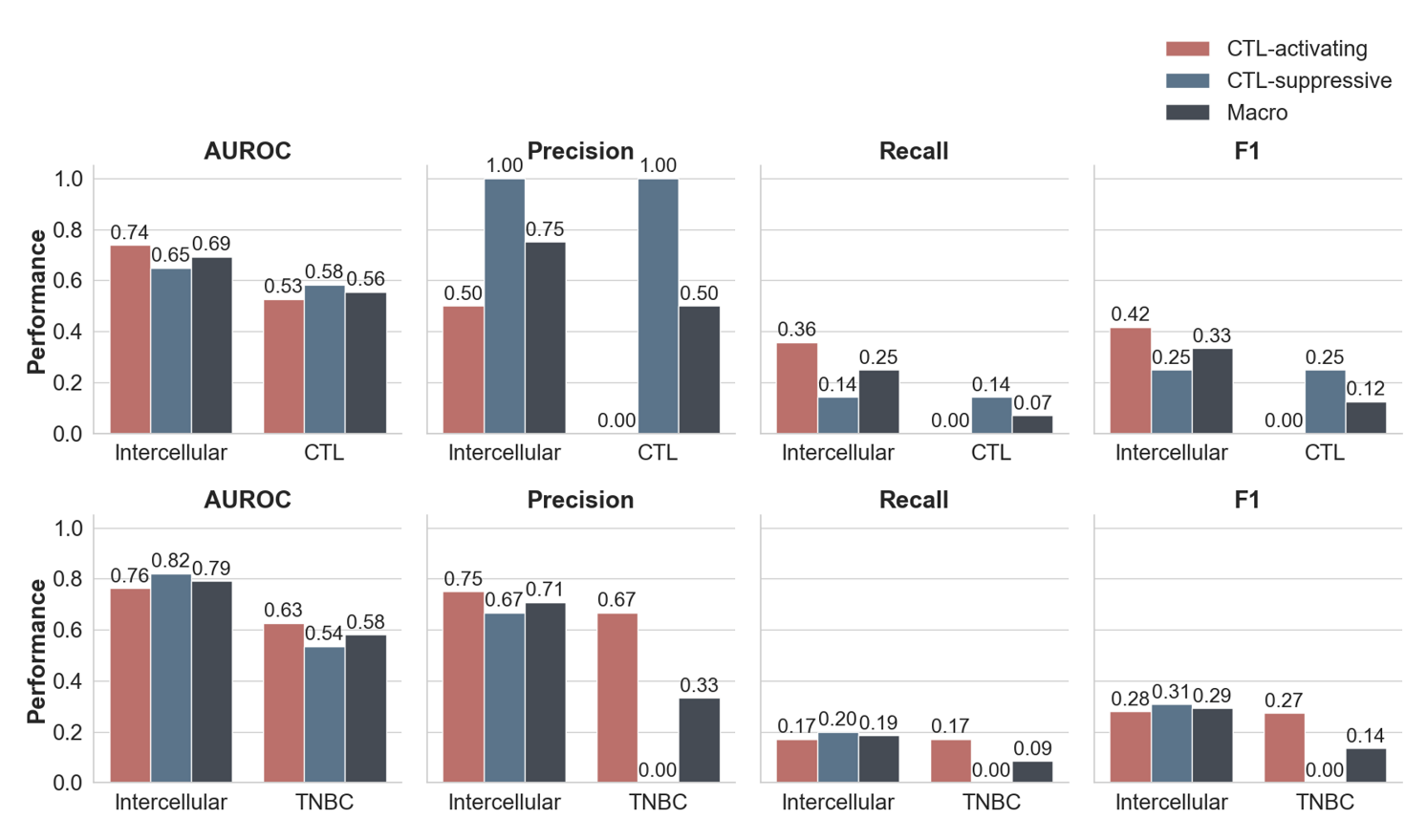


Supplementary Figure 6. Performance comparison between gene-level intercellular and single CRISPR screens. The gene-level intercellular CRISPR screen outperforms single CRISPR screens in all evaluation metrics. AUROC: area under the receiver operating characteristic curve, CTL: cytotoxic T lymphocyte, TNBC: triple-negative breast cancer.

## Supplementary Tables

**Supplementary Table 1**. The statistics of approved drugs and their targets. IO: immuno-oncology.

**Supplementary Table 2**. Intercellular normZ scores and corresponding FDRs for all intercellular interactions. FDR: false discovery rate, CTL: cytotoxic T lymphocyte, TNBC: triple-negative breast cancer.

**Supplementary Table 3**. Gold standard dataset of 38 intercellular interactions targeted by approved drugs or phase III clinical trial drug candidates for immunotherapy. TNBC: triple-negative breast cancer, CTL: cytotoxic T lymphocyte.
